# Bioinformatics Combined With Biological Experiments to Identify the Pathogenetic Link of Type 2 Diabetes for Breast Cancer

**DOI:** 10.1002/cam4.70759

**Published:** 2025-04-09

**Authors:** Xin Bao, Zhirui Zeng, Wenjing Tang, Dahuan Li, Xianrui Fan, Kang Chen, Yongkang Wang, Weijie Ai, Qian Yang, Shu Liu, Tengxiang Chen

**Affiliations:** ^1^ Engineering Research Center of Chronic Disease Diagnosis and Treatment, School of Basic Medicine Guizhou Medical University Guiyang China; ^2^ School of Imaging Guizhou Medical University Guiyang China; ^3^ Department of Breast Surgery Affiliated Hospital of Guizhou Medical University Guiyang China

**Keywords:** bioinformatics, biological experiments, breast cancer, pathogenetic link, type 2 diabetes

## Abstract

**Background:**

Type 2 diabetes mellitus (T2DM) constitutes a significant risk factor for breast cancer (BC), with affected women exhibiting a two‐ to three‐fold increased likelihood of developing BC. Furthermore, women diagnosed with both BC and T2DM tend to experience poorer prognoses and exhibit greater resistance to various treatments compared to their non‐diabetic counterparts. Consequently, elucidating the comorbidities associated with T2DM and BC is instrumental in enhancing the diagnostic and therapeutic strategies for BC.

**Methods:**

A series of bioinformatics methods including weighted gene co‐expression network analysis (WGCNA), differentially expressed gene (DEG) analysis, machine learning, and single‐cell sequencing analysis were used to identify the pathogenetic molecules of T2DM for BC. Biological experiments including CCK‐8, colony formation, wound healing, transwell assay, immunohistochemistry, and immunofluorescence were performed to determine the molecule effect.

**Results:**

By conducting WGCNA and DEG analysis on the profiles of T2DM (GSE25724 and GSE20966) and the TCGA cohort of BC, we identified a total of 27 common hub genes shared between T2DM and BC. These genes were significantly enriched in pathways related to cell differentiation, cellular developmental processes, focal adhesion, and the MAPK signaling pathway. Notably, among these 27 genes, *CCNB2*, *XRCC2*, and *CENPI* were associated with poor prognosis in BC. Moreover, single‐cell RNA sequencing analysis revealed that *CCNB2*, *XRCC2*, and *CENPI* are enriched in cancer cells within BC tissues. Additionally, we observed that CCNB2, XRCC2, and CENPI were elevated in BC tissues provided by patients with a diabetes history and associated with KI67 expression. Hyperglycemia treatment elevated the expression levels of CCNB2, XRCC2, and CENPI in BC cells, which correlated with increased cell proliferation and mobility. Conversely, the knockdown of these genes partially mitigated the pro‐proliferative and pro‐migratory effects induced by hyperglycemia in BC cells.

**Conclusion:**

Our findings suggested that CCNB2, XRCC2, and CENPI may serve as key pathogenic mediators linking T2DM and BC. Targeting these molecules could potentially attenuate the adverse impacts of T2DM on BC progression.

## 1 Introduction

Breast cancer (BC) represents a prevalent malignant neoplasm globally, with metastasis being the principal cause of mortality among affected individuals [1]. The prognosis for BC patients is significantly influenced by the pathological stage of the disease, with a 5‐year survival rate reaching up to 85% for those diagnosed at an early stage, compared to less than 30% for those with advanced metastatic disease [2, 3]. Identified risk factors for BC encompass obesity, diabetes, nulliparity, and lack of breastfeeding [4, 5]. Research indicated that type 2 diabetes mellitus (T2DM) constitutes a significant risk factor for BC. Women diagnosed with T2DM exhibit a 2‐ to 3‐fold increased risk of developing BC compared to women without diabetes [6]. Furthermore, women with BC who also have T2DM tend to have a poorer prognosis and demonstrate resistance to various treatments [7, 8]. Consequently, elucidating the “cross‐talk” mechanisms between T2DM and BC is crucial for improving the diagnosis and treatment of BC.

Diabetes mellitus is a chronic metabolic disorder characterized by elevated blood glucose levels [9]. China ranks among the countries with the highest prevalence of diabetes globally. Diabetes complications and associated deaths have contributed to annual diabetes‐related health care expenditures in China, which ranks second in the world for these expenditures at $165.3 billion [10]. Literature reports suggest that elevated blood glucose levels and transforming growth factor levels in diabetic patients may exacerbate certain complications associated with the disease [11, 12]. Intriguingly, prior studies identified blood glucose and transforming growth factors as significant microenvironmental factors that facilitated the progression of BC [13, 14]. Nonetheless, the mechanisms by which these factors exacerbate complications and advance BC in patients with diabetes remain poorly understood. Consequently, it is imperative to investigate the molecular interactions between T2DM and BC to enhance therapeutic strategies for BC.

Bioinformatics is an interdisciplinary field that encompasses the acquisition, processing, storage, distribution, analysis, and interpretation of biological information. It integrates tools from mathematics, computer science, and biology to elucidate and comprehend the biological significance embedded within extensive datasets. This discipline is instrumental in identifying and evaluating disease‐specific biomarkers, predicting and assessing disease onset and progression, severity, dynamic changes, treatment sensitivity and resistance, as well as patient prognosis [15, 16]. For example, Ali Golestan's research [17] identified *CACNG4*, *PKMYT1*, *EPYC*, and *CHRNA6* as potential biomarkers for BC diagnosis, with *PKMYT1* also demonstrating prognostic significance through DEG analysis. Min Liu et al. [18] performed bioinformatics analysis for miRNA expression data from the TCGA and GEO databases, revealing that plasma exosomal hsa‐miR‐21‐5p could serve as a biomarker for BC diagnosis. Xie et al. [19] employed multi‐omics analysis on TCGA data and identified that glycogen synthase 1 was a key target for BC, especially for triple‐negative BC. Nonetheless, due to tumor heterogeneity, low response rates, relapse, and drug resistance, there remains a pressing need to identify novel biomarkers that could enhance the diagnosis and treatment of BC.

In this study, bioinformatics analyses of profiles from T2DM and BC identified CCNB2, XRCC2, and CENPI as key pathogenic mediators linking T2DM and BC. Hyperglycemia was observed to elevate the expression levels of CCNB2, XRCC2, and CENPI in BC cells, which were associated with increased cell proliferation and mobility. Knockdown of these genes partially mitigated the pro‐proliferative and pro‐migratory effects induced by hyperglycemia in BC cells. Consequently, targeting these molecules may potentially attenuate the adverse impacts of T2DM on BC progression.

## 2 Materials and Methods

### 2.1 Download and Pre‐Process of Profiles of T2DM and BC

We downloaded T2DM (GSE25724 and GSE20966) profiles from the Gene Expression Omnibus (GEO) database (https://www.ncbi.nlm.nih.gov/gds/). The GSE25724 dataset [20], based on the GPL96 platform, comprises 7 non‐diabetic islet samples and 6 T2DM islet samples. The GSE20966 dataset [21], based on the GPL1352 platform, consisted of 10 non‐diabetic samples and 10 diabetic samples. Initially, we utilized the R package inSilicoMerging (version: 1.0.8) to integrate the datasets. Subsequently, we applied empirical Bayes methods [22, 23] to eliminate batch effects, resulting in a matrix containing 2579 genes after batch effect correction. We obtained the dataset of BC from the University of California, Santa Cruz (UCSC) Xena platform (https://xena.ucsc.edu/). Columns (samples) and rows (genes) with more than 50% missing values were excluded from the analysis. Subsequently, the impute.knn function from the R package ‘impute’ (Version:1.78.0) was employed to address the remaining missing values, with the number of neighbors specified for the imputation process. For genes with duplicate entries, the median value was computed and utilized.

### 2.2 Differentially Expressed Gene Analysis

We employed the “limma” R package (Version: 3.60.4) for data quality control, processing, and statistical analysis. The gene expression profiles were standardized using the robust multi‐array average method. Differentially expressed genes (DEGs)were discovered between BC tissues and non‐tumor tissues with a false discovery rate(FDR)below 0.05 and a fold change exceeding 2. Similarly, DEGs between tissues from patients with T2DM and healthy individuals were identified with an FDR of less than 0.05 and an absolute fold change greater than 1.2.

### 2.3 Identification of Genes Related to T2DM and BC Through Weighted Gene Co‐Expression Network Analysis

We used the weighted gene co‐expression network analysis (WGCNA) package (Version:1.72–5) to develop a gene co‐expression network and identify genes associated with specific phenotypic modules, such as those related to the T2DM state and BC state. Initially, we performed hclust to check whether outliers existed between the samples. Then, we computed the Pearson correlation coefficients for all gene pairs to construct an adjacency matrix, thereby enhancing strong gene correlations and penalizing weaker associations. Subsequently, the adjacency matrix was converted into a topological overlap matrix (TOM) and average linkage hierarchical clustering was conducted to group similar gene classes into distinct modules. Third, gene significance (GS) and module membership (MM) were computed to correlate the identified modules with clinical features. Finally, the network of co‐expression modules was visualized. Genes correlating with these modules were then utilized for the later stages of the analysis.

### 2.4 Hub‐Related Genes Screening and the Correlation Analysis

To utilize the co‐expression network analysis, the “VennDiagram” R package (Version: 1.7.3) was employed to intersect highly module‐related genes with DEGs, thereby identifying differentially expressed trait‐related module genes (also named hub genes) associated with T2DM and BC. Subsequently, the expression profiles of hub genes within the BC and T2DM datasets were extracted. The “heatmap” R package (Version:1.0.12) was then utilized to generate a heatmap of gene expression, and the “corrplot” R package (Version:0.92) was used to calculate the correlation between gene transcript levels.

### 2.5 Gene Ontology Functional Annotation and Kyoto Encyclopedia of Genes and Genomes Analysis

To acquire deeper insights into the biological functions of hub genes associated with T2DM and BC, we employed Gene Ontology (GO) and Kyoto Encyclopedia of Genes and Genomes (KEGG) pathway analyses utilizing the “clusterProfiler” R package (Version:4.12.2). Significance thresholds were established at *p* < 0.05.

### 2.6 Machine Learning of Hub Genes

The hub genes' prognostic values were examined using a range of machine learning techniques. First, KM plot analysis was employed to analyze the relationship between expression of hub genes. Support vector machine recursive feature elimination (SVM‐RFE) is a feature selection method which recursively trains the SVM model to assess the importance of each feature, gradually eliminating features with less contribution to classification, until only the most discriminative features remain [24]. Least absolute shrinkage and election operator (LASSO) is a regularization method used for regression analysis. It works by shrinking some of the regression coefficients, making them zero, thereby performing feature selection and improving the model's generalization ability [25]. Boruta random forest is a feature selection method based on random forests. It compares the original features with their randomly shuffled versions and iterates repeatedly, ultimately retaining the features that have a significant impact on the classification task [26]. Then, SVM‐RFE, LASSO regression analysis, and Boruta random forest were conducted in sequence by using the R package ‘rms’ (version: 6.8.2), ‘lfa’ (version: 2.5.0), ‘glmnet’ (version: 4.1.8), ‘Boruta’ (version: 8.0.0), ‘random Forest’ (version: 3.3.1), and ‘sigFeature’ (version: 3.19).

### 2.7 Single‐Cell RNA Sequencing Analysis of BC Tissues

We conducted single‐cell sequencing analysis utilizing the ArrayExpress matrix (EMTAB8107) (http://tisch.comp‐genomics.org/). Initially, we calculated the percentage of mitochondrial genes within cells using the “Percentage Feature” package (Version: 5.0.3). Cells were then screened using the following criteria: minimum mitochondrial gene percentage of 20%, minimum red blood cell gene percentage of 20%, gene expression counts ≥ 1000, and gene expression features falling within the range of 200 to 10,000. Next, we assessed the correlation between gene features and sequencing depth to evaluate data reliability. Afterward, we chose 2000 genes with high variability for additional examination. We set the number of principal components (PCs) to 15 to facilitate the identification of cell clusters. Subsequently, cell clusters were visualized using a UMAP plot generated by the “UMAP” R package (Version:0.2.10.0). Third, cell annotation was conducted using the “singleR” R package(Version 2.6.0). Finally, we assessed the expression of biomarker genes within the clusters and employed the “VlnPlot” package (Version:5.0.3) to illustrate the expression levels of CCNB2, XRCC2, and CENPI in each cell type.

### 2.8 Tissue Collection and Immunohistochemical Analysis

A total of 60 BC tissues were collected from the Affiliated Hospital of Guizhou Medical University, following approval by the Human Ethics Committee of the same institution (Approval number: 2024–187). A written informed consent was obtained for each patient. Among them, 42 BC tissues were provided from patients without a diabetes history, while 18 of them were provided from patients with a diabetes history. The BC tissues were fixed, dehydrated, encased in paraffin, and then cut into 2‐μm slices. These sections were then deparaffinized and re‐hydrated using xylene (Sinopharm, Beijing, China) and a descending alcohol series (Sinopharm, Beijing, China), respectively. After antigen retrieval using 100 mM sodium citrate (ZSGB‐BIO, Beijing, China), the tissue sections were blocked with 3% hydrogen peroxide (Sinopharm, Beijing, China) and 5% bovine serum albumin (Boster, Wuhan, China) at room temperature. The sections were incubated with an anti‐XRCC2 antibody (1:200; cat no. A1800, Abconal, Wuhan, China), anti‐CCNB2 (1:200; cat no. CCNB2, Proteintech, Wuhan, China), anti‐CENPI (1:100; cat no. DF2311, Affinity Biosciences, Beijing, China), and KI67 (1:2000; cat no. 27309‐1‐AP, Proteintech, Wuhan, China) overnight at 4°C subsequently. Following the incubation period, the sections were exposed to a secondary antibody for 2 h. We then stained the slides using a Cell and Tissue Staining HRP‐3,3′‐diaminobenzidine kit (ZSGB‐BIO, Beijing, China), followed by nuclear counterstaining with 0.2% hematoxylin (ZSGB‐BIO, Beijing, China) for 1 min. We visualized the staining using a light microscope at a magnification of × 200 and × 400.

### 2.9 Cell Culture and Transfection

The BC cells (MCF‐7 and MDA‐MB‐231) were purchased from Procell (Wuhan, China) and cultured in Dulbecco's modified Eagle medium (DMEM) with 10% fetal bovine serum (FBS) at 37°C in a 5% CO_2_ incubator. The cells in the logarithmic phase were chosen for functional experiments. The sugar concentration of the normal glucose group medium was 5.5 mmol/L. The sugar concentration of the high glucose group 1 medium was 25 mmol/L, and the sugar concentration of the high glucose group 2 medium was 55 mmol/L according to previous studies [27, 28]. The additional glucose added is dextrose (cat no. A100188‐0500 Diamond, Taiwan, China). siRNAs targeting CENPI, CCNB2, and XRCC2, as well as negative control(NC)siRNAs, were procured from iGeneBio in Beijing, China. CENPI, CCNB2 and XRCC2 knockdown was realized by transfecting cells with indicated siRNAs using Lipofectamine 3000 reagent (Invitrogen, USA) as directed by the manufacturer's instructions.

### 2.10 Western Blot

MCF‐7 and MDA‐MB‐231 cell's total protein was extracted using RIPA lysis buffer (Solarbio, Wuhan, China). Protein concentrations were measured using an Enhanced BCA kit from Beyotime located in Nanjing, China. Equal amounts of protein(30 μg)were separated using 10%SDS‐PAGEand subsequently transferred onto a PVDF membrane from Millipore, MA, USA. Subsequently, blocking with 5% BSA, the membranes were incubated overnight at 4°C with primary antibodies, including anti‐CCNB2 (1:1000, cat no. A7956 ABclonal Technology Inc., Wuhan, China), anti‐XRCC2 (1:500, cat no. A1800 ABclonal Technology Inc., Wuhan, China), anti‐CENPI(1:1000, cat no. YN1576 ImmunoWay Biotechnology Company, USA), and anti‐β‐actin (1:1000, cat no. AC006 ABclonal Technology Inc., Wuhan, China). Then, the membranes were incubated with HRP‐conjugated secondary antibodies for 2 h at room temperature and subsequently washed three times with TBST. Protein bands were detected using an ECL reagent from Beyotime in the Bio‐Rad system and then scanned and quantified using ImageJ software from the National Institutes of Health. The Relative expression of the target proteins was normalized to β‐actin.

### 2.11 Cell Counting Kit‐8 and Colony Formation

The proliferative abilities of MCF‐7 and MDA‐MB‐231 cells were evaluated utilizing the Cell Counting Kit‐8 (CCK‐8) assay and colony formation assay. Cells were seeded at a density of 2000 cells per well in 96‐well plates and incubated overnight for the CCK‐8 assay. Cell growth was monitored at various time points using the CCK‐8 kit (C6005M, UElandy, China), and the absorbance of each well at 450 nm was measured after a 2‐h incubation using an iMark microplate reader (Bio‐Rad Laboratories). In colony formation assays, around 500 cells were plated into individual wells of six‐well plates. Upon the emergence of colonies, the cells were sequentially fixed with 4% methanol and stained using crystal violet, respectively.

### 2.12 Wound Healing Assay

MCF‐7 and MDA‐MB‐231 cells(6 × 105 cells per well)were grown in six‐well plates until they reached about 95%confluency. In the monolayer cultures, wounds were created with a 200‐μL pipette tip. Cells were detached by washing twice with PBS before being subjected to the specified treatments. Mitomycin C was administered at a concentration of 1 μM to inhibit cell proliferation. The wound healing process was monitored and documented over a period of 0 to 24 h using an optical microscope at 40× magnification.

### 2.13 Transwell Invasion Assay

Invasion assays were performed by 24‐well transwell chambers precoated with Matrigel (Corning, USA). 2 × 10^4^ MCF‐7 and MDA‐MB‐231 cells were seeded into the upper chambers of the transwell system in serum‐free DMEM medium approximately, while the lower chambers were added with DMEM containing 10% FBS. After a 24‐h incubation period, the cells that had penetrated through the Matrigel were fixed with 4% methanol, stained with crystal violet, and subsequently photographed and quantified using an inverted microscope.

### 2.14 Immunofluorescence

MDA‐MB‐231 cells were seeded onto glass cover slides and incubated at 37°C overnight. Following fixation with 4% formaldehyde (Boster, Wuhan, China), MDA‐MB‐231 cells were permeabilized with 0.3% Triton X‐100 and blocked using 5% BSA (Boster, Wuhan, China). The cells were then incubated with primary antibodies (1:200 dilution) including E‐cadherin (20874‐1‐AP, Proteintech, Wuhan, China) and vimentin (60330‐1‐Ig, Proteintech, Wuhan, China) at 4°C overnight. Afterward, they were incubated with Alexa Fluor 594‐conjugated secondary antibodies (1:200 dilution) for 1 h at room temperature, followed by DAPI nuclear staining for 10 min at room temperature. Images were captured using a Leica DM2500 fluorescence microscope.

### 2.15 Data Statistics

The data were analyzed using SPSS (version 19.0). Differences between the two groups were assessed using an unpaired t‐test, while differences among multiple groups were evaluated using one‐way analysis of variance (ANOVA). Statistical significance was set at *p* < 0.05.

## 3 Results

### 3.1 DEGs Analysis for T2DM and BC

**FIGURE 1:**
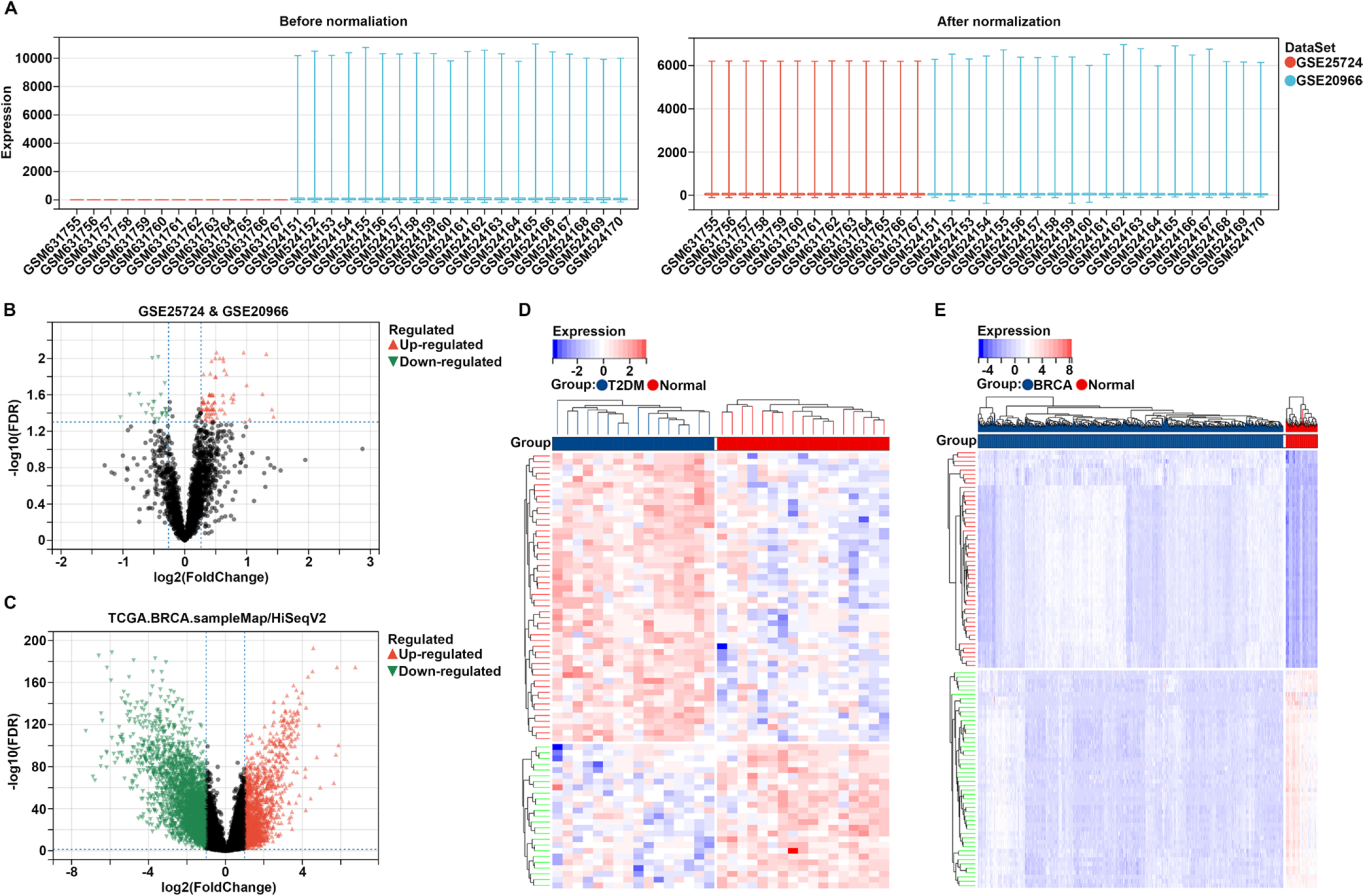
DEGs analysis for T2DM and BC. (A) Normalization and merging the profiles of T2DM (GSE25724 and GSE20966) downloaded from the GEO database. (B) DEGs analysis identified the DEGs in T2DM. (C) DEGs identified in BC after the profile normalization. (D, E) DEGs in T2DM and BC were exhibited in heatmaps respectively.

Two profiles of T2DM, including GSE25724 and GSE20966, were obtained from the GEO database. Prior to merging, these profiles were normalized using the robust multi‐array average method (Figure 1A). DEGs analysis identified a total of 109 DEGs in T2DM, with 84 genes being upregulated and 25 genes downregulated (Figure 1B). Similarly, following normalization of the BC profile from the TCGA cohort, a total of 4227 DEGs were identified in BC tissues compared to non‐tumor tissues, comprising 1413 upregulated genes and 2814 downregulated genes (Figure 1C). DEGs in T2DM (Figure 1D) and BC (Figure 1E) were exhibited in heatmap, respectively, and used for further analysis.

### 3.2 Construction of WGCNA for T2DM

**FIGURE 2:**
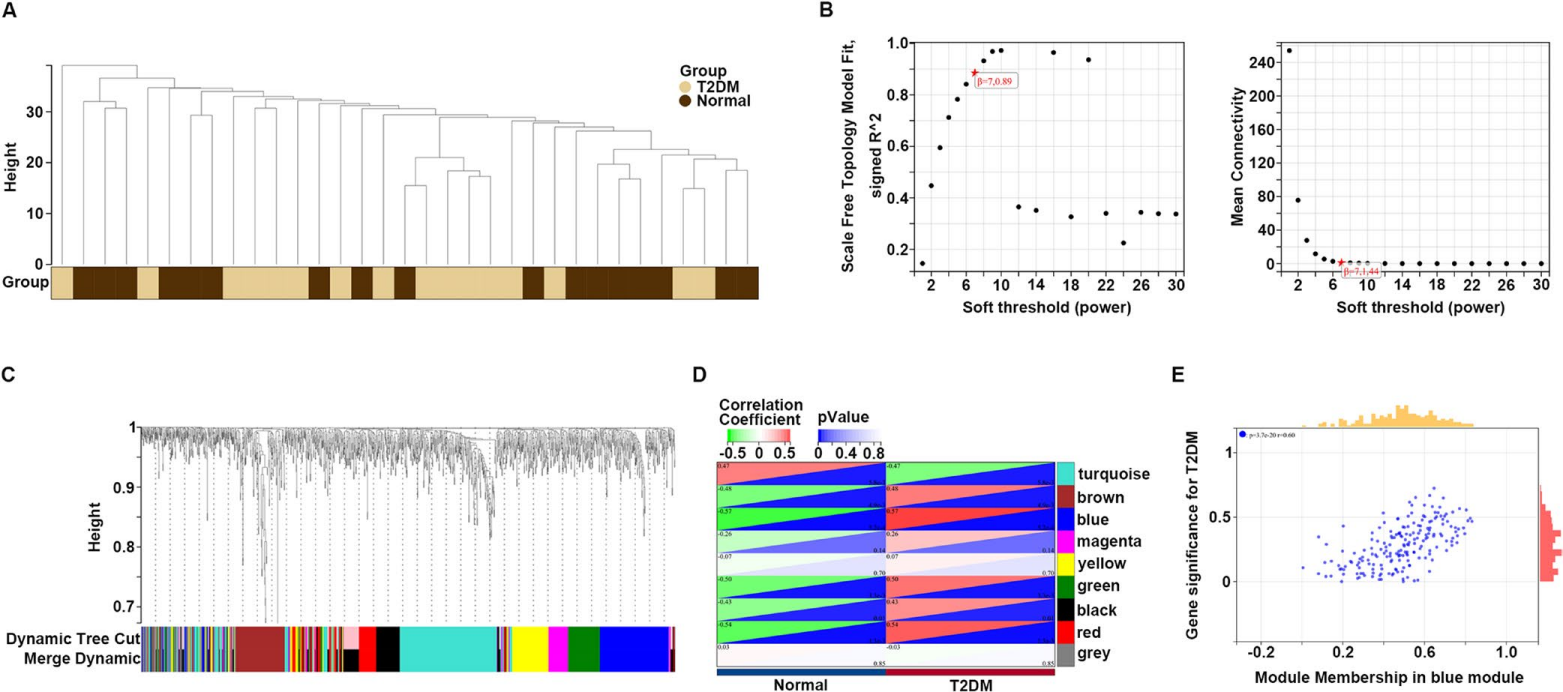
Construction of WGCNA for T2DM. (A) Elimination of outliers and sample filtering. (B) Determination of soft threshold. (C) Dendrogram of all expressed genes clustered based on a dissimilarity measure (1‐TOM). (D) Heatmap of the correlation between module eigengenes and clinical traits in T2DM. (E) Scatter plots of the degree and *p*‐value of Cox regression in the blue module. The *x*‐axis indicated the degree of regression, the *y*‐axis indicated the gene significance. Each dot represent gene.

Prior to conducting WGCNA, hclust was performed on the merged sample profiles from GSE25724 and GSE20966, revealing no outlier samples with a cut height greater than 40 (Figure 2A). To ensure the construction of a scale‐free network, we employed empirical analysis to determine the appropriate soft threshold. The results indicated that a soft power of 7 satisfied the criteria of a topological model fitting index ≥ 0.85 and mean connectivity close to 0 (Figure 2B). After selecting the appropriate soft power, all genes were clustered into eight co‐expression modules: turquoise, brown, blue, magenta, yellow, green, black, and red. Genes without co‐expression relationships were grouped into gray modules (Figure 2C,D). Subsequently, we examined the relationship between these gene modules and clinical characteristics, revealing that genes within the turquoise, red, green, brown, and blue modules were associated with T2DM with a strong co‐relationship score (|*R*| ≥ 0.45, *p* < 0.05) (Figure 2D). Furthermore, by calculating the GS and MM within these modules, we observed that all GS values in the turquoise (Figure S1A), red (Figure S1B), green (Figure S1C), brown (Figure S1D), and blue (Figure 2E) modules were positively correlated with their respective MM values. Notably, the genes in the blue module exhibited the strongest correlation between GS and MM (correlation = 0.60) (Figure 2E). Therefore, genes within these five modules exhibiting |MM| ≥ 0.5 and |GS| ≥ 0.5 were designated as core genes of T2DM and subjected to further analysis (Table S1).

### 3.3 Construction of WGCNA for BC

**FIGURE 3:**
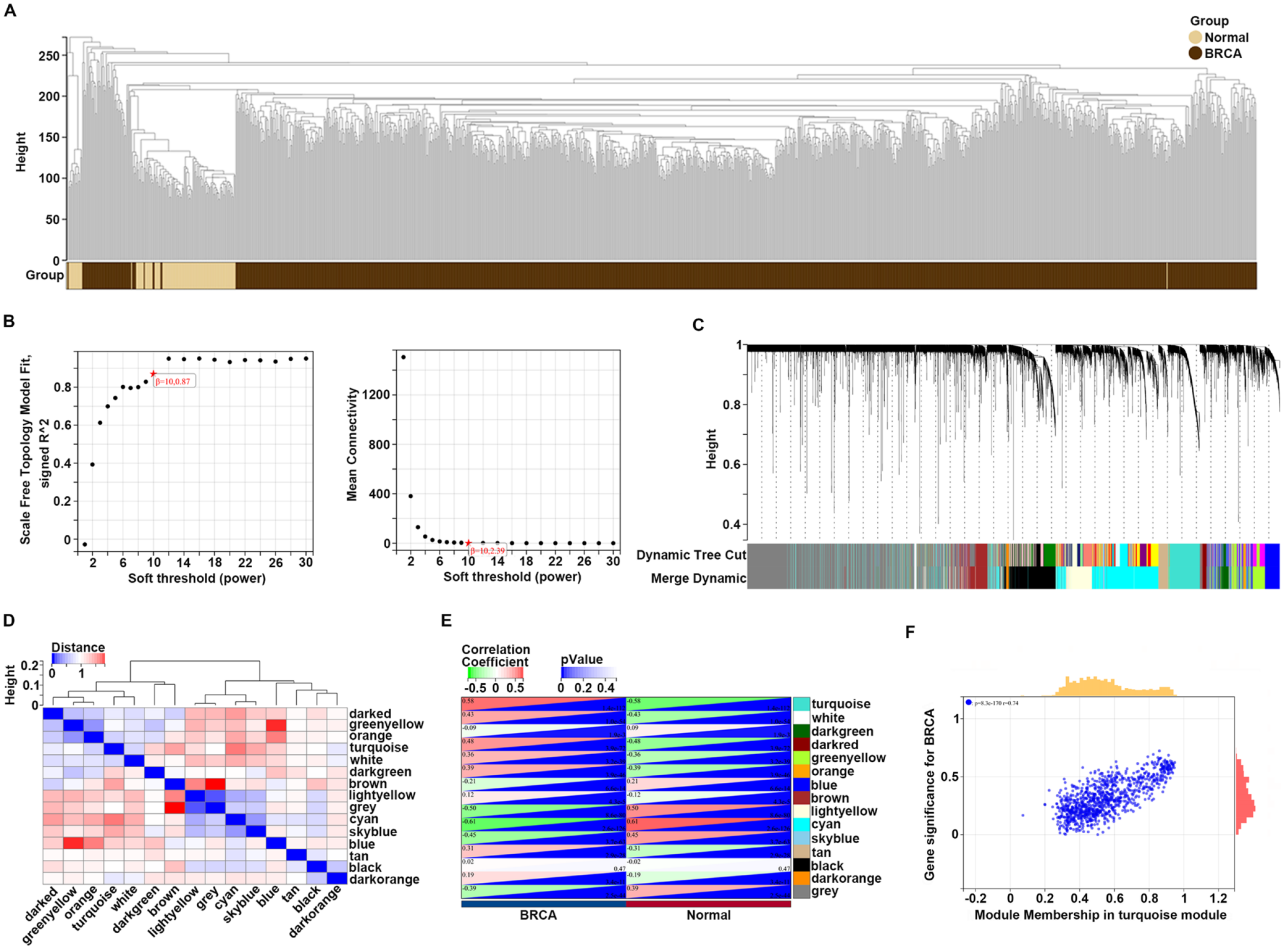
Construction of WGCNA for BC. (A) Elimination of outliers and sample filtering. (B) Determination of soft threshold. (C) Dendrogram of all expressed genes clustered based on a dissimilarity measure (1‐TOM). (D) Heatmap of the correlation between module eigengenes. (E) Heatmap of the correlation between module eigengenes and clinical traits in BC. (F) Scatter plots of the degree and *p*‐value of Cox regression in the turquoise module. The *x*‐axis indicated the degree of regression, the *y*‐axis indicated the gene significance. Each dot represented gene.

Subsequently, the same methodology was employed to construct the WGCNA network for the BC matrix. Hclust was performed on the profiles, revealing no outliers at a cut height greater than 270 (Figure 3A). Empirical analysis was then conducted, determining that a soft power of 10 met the criteria of a topological model fitting index ≥ 0.85 and mean connectivity approximating 0 (Figure 3B). A total of 14 gene co‐expression modules were identified, whereas genes lacking co‐expression relationships were grouped into gray modules (Figure 3C). Among these, the most robust positive correlation was observed between the green‐yellow module signature and the blue module signature (Figure 3D). Furthermore, the signatures of the turquoise, dark red, light yellow, cyan, and sky blue modules were found to be significantly associated with the BC state (|*R*| ≥ 0.45, *p* < 0.05) (Figure 3E). Similarly, by calculating the GS and MM within these modules, we observed that all GS values in the turquoise (Figure 3F), sky blue (Figure S2A), light yellow (Figure S2B), dark red (Figure S2C), and cyan (Figure S2D) modules were correlated with their respective MM values. Notably, the turquoise module demonstrated the highest co‐expression relationship between GS and MM (Figure 3F). Consequently, genes within these modules exhibiting |MM| ≥ 0.5 and |GS| ≥ 0.5 were designated as core module genes of BC and were selected for further analysis (Table S2).

### 3.4 Hub Gene Selection and Enrichment Analysis

**FIGURE 4:**
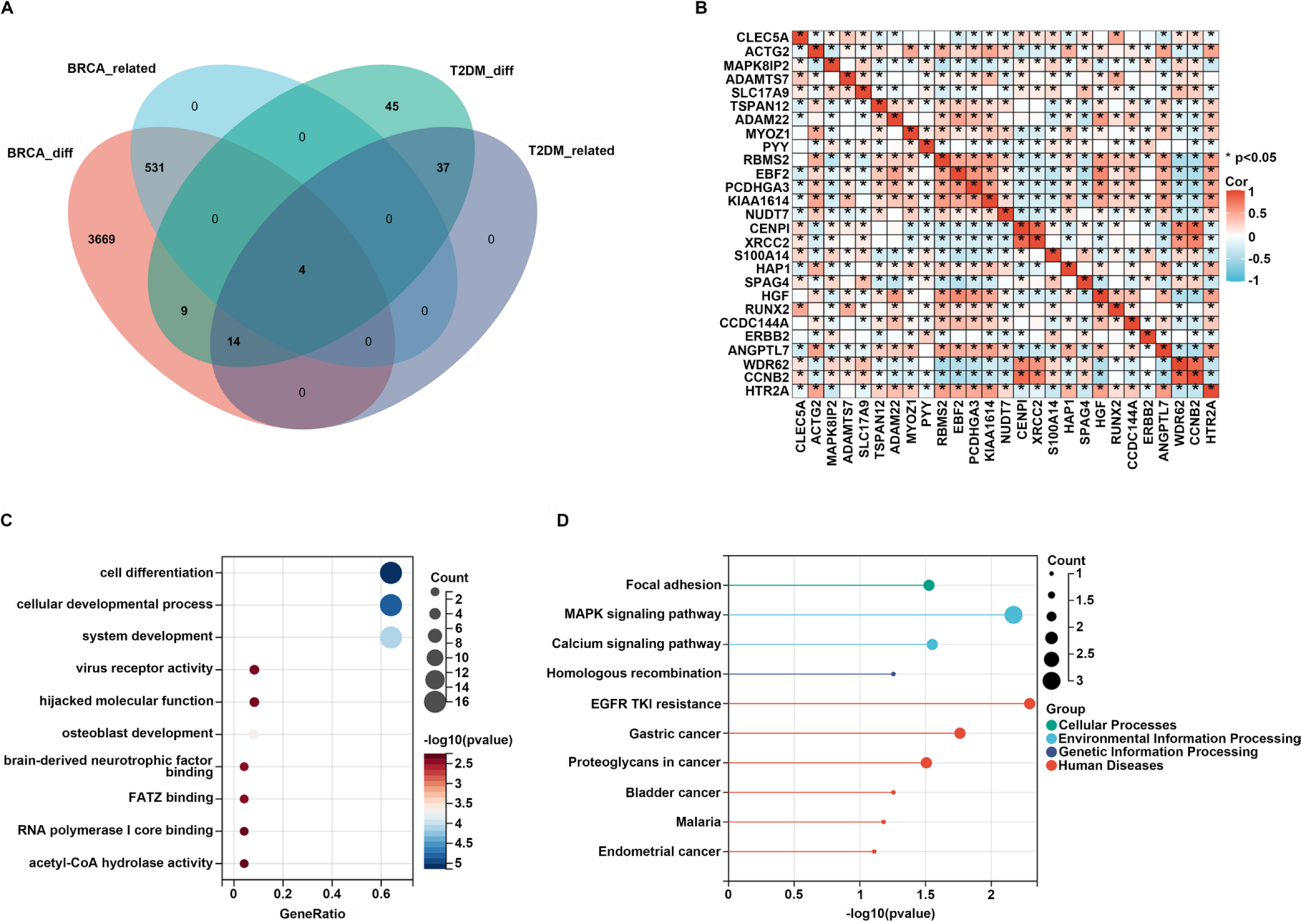
Hub gene selection and enrichment analysis. (A) Venn diagram of the shared genes between T2DM and two BC. (B) Spearman correlation analysis of 27 shared genes of T2DM and BC. (C) GO biological process of the shared genes. (D) KEGG analysis of the shared genes. *, *p* < 0.05.

To identify the pathogenetic link molecules between T2DM and BC, we conducted an intersection analysis of the DEGs and module core genes associated with T2DM or BC. Our analysis revealed a total of 27 genes associated with BC that were differentially expressed in tissues from T2DM patients and/or within modules related to the T2DM state (Figure 4A). These 27 genes were set as hub genes between T2DM and BC. Subsequently, we extracted the expression profiles of these 27 hub genes and constructed a correlation heatmap based on the matrix profile. The results indicated that the correlations among most gene pairs were strong (Figure 4B). Furthermore, we conducted a biological enrichment analysis of the aforementioned genes. GO functional annotation revealed that these genes are predominantly associated with cell differentiation, cellular developmental processes, and system development (Figure 4C). KEGG pathway analysis indicated significant enrichment in focal adhesion, MAPK signaling pathway, and calcium signaling pathway (Figure 4D).

### 3.5 CCNB2, XRCC2, and CENPI Were Found to Be Associated With Poor Prognosis in BC

**FIGURE 5:**
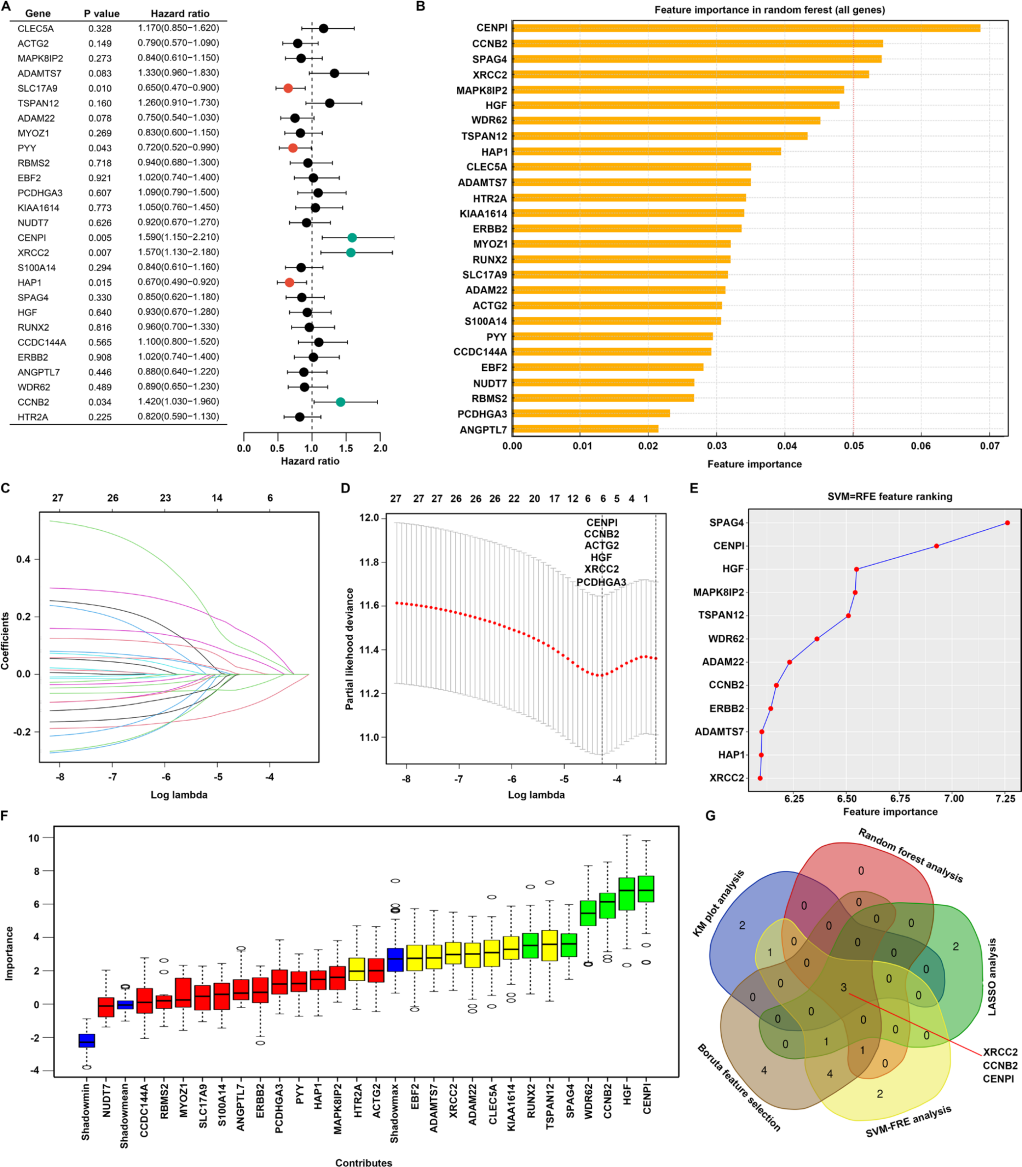
CCNB2, XRCC2, and CENPI were found to be associated with poor prognosis in BC. (A) Kaplan–Meier plot analysis for the 27 hub genes. (B) Random forest analysis for the 27 hub genes. (C, D) LASSO analysis for the 27 hub genes. (E) SVM‐RFE model for the 27 hub genes. (F) Boruta random forest analysis for the 27 hub genes. (G) Integration of machine learning methods indicated CCNB2, XRCC2, and CENPI were the most important prognostic biomarkers for BC.

Subsequently, we evaluated the prognostic significance of 27 hub genes in BC utilizing a range of machine learning techniques. Kaplan–Meier plot analysis revealed that among these genes, SLC17A9, PYY, and HAP1 were associated with favorable outcomes in BC, whereas CENPI, XRCC2, and CCNB2 were linked to unfavorable outcomes (Figure 5A). Furthermore, random forest analysis identified CENPI, CCNB2, SPAG4, and XRCC2 as having substantial prognostic importance for BC patients, each with an importance score exceeding 0.05 (Figure 5B). LASSO analysis was conducted, revealing that CENPI, CCNB2, ACTG2, HGF, XRCC2, and PCDHGA3 are key genes associated with BC prognosis (Figure 5C,D). Additionally, the SVM‐RFE model identified SPAG4, CENPI, HGF, MAPK8IP2, WDR62, ADAM22, CCNB2, ERBB2, ADAMTS7, HAP1, and XRCC2 as prognostic genes among the 27 hub genes for BC (Figure 5E). Furthermore, Boruta random forest analysis highlighted CENPI, XRCC2, HGF, CCNB2, WDR62, SPAG4, TSPAN12, RUNX2, KIAA1614, CLEC5A, ADAM22, ADAMTS7, and EBF2 as significant contributors to the prognosis of BC patients (Figure 5F). The integration of these machine learning techniques collectively demonstrated that XRCC2, CENPI, and CCNB2 serve as prognostic biomarkers for BC (Figure 5G). Importantly, we observed that the expression levels of CCNB2, XRCC2, and CENPI were elevated in both T2DM and BC tissues (Table S3). Consequently, our research concentrated on these three pivotal genes.

### 3.6 CCNB2, XRCC2, and CENPI Were Mostly Enriched in Cancer Cells in BC Tissues

**FIGURE 6:**
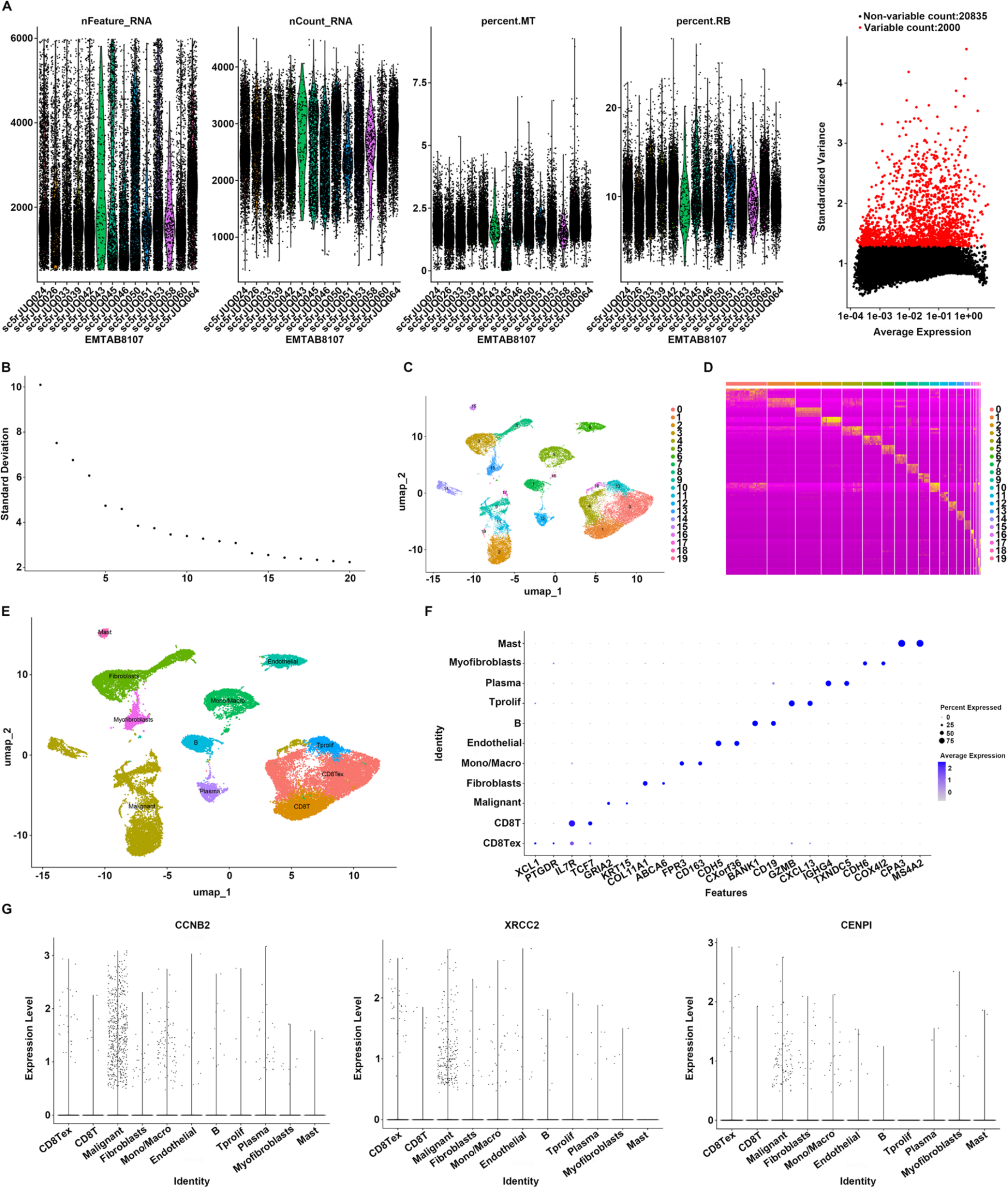
CCNB2, XRCC2, and CENPI were mostly enriched in cancer cells in BC tissues. (A) Cells exhibiting low expression levels were excluded based on predefined filtering criteria. (B) PCA was performed using 20 principal components to facilitate cell clustering. (C, D) Identification of 20 distinct cell clusters. (E) Cell clusters annotated into 11 distinct cell types. (F) Biomarkers for these cell types were exhibited. (G) CCNB2, XRCC2, and CENPI were predominantly expressed in the malignant‐annotated cell cluster, with significantly lower expression levels observed in other cell clusters.

Furthermore, we investigated the cell localization of the three hub genes by conducting single‐cell RNA (scRNA)‐seq analysis on the BC tissue dataset EMTAB8107. Initially, cells exhibiting low expression levels were excluded based on predefined filtering criteria (Figure 6A). Subsequently, the 2000 most highly variable genes within these cells were selected for further analysis (Figure 6A). PCA was performed using 20 PCs to facilitate cell clustering (Figure 6B), resulting in the identification of 20 distinct cell clusters (Figure 6C,D). These cell clusters were then annotated into 11 distinct cell types, including malignant cells, mast cells, myofibroblasts, plasma cells, proliferation T cells (Tprolif), B cells, endothelial cells, monocyte/macrophage cells, fibroblasts, CD8 T cells, and exhausted CD8 T cells (Figure 6E). Biomarkers for these cell types were exhibited (Figure 6F). Furthermore, our analysis revealed that the three key genes, including CCNB2, XRCC2, and CENPI, were predominantly expressed in the malignant‐annotated cell cluster, with significantly lower expression levels observed in other cell clusters (Figure 6G). This differential expression pattern suggested that these three key genes are critically involved in the pathogenesis and progression of BC.

### 3.7 Knockdown of CCNB2, XRCC2, and CENPI Potentially Attenuates the Adverse Impacts of T2DM on BC Progression

**FIGURE 7:**
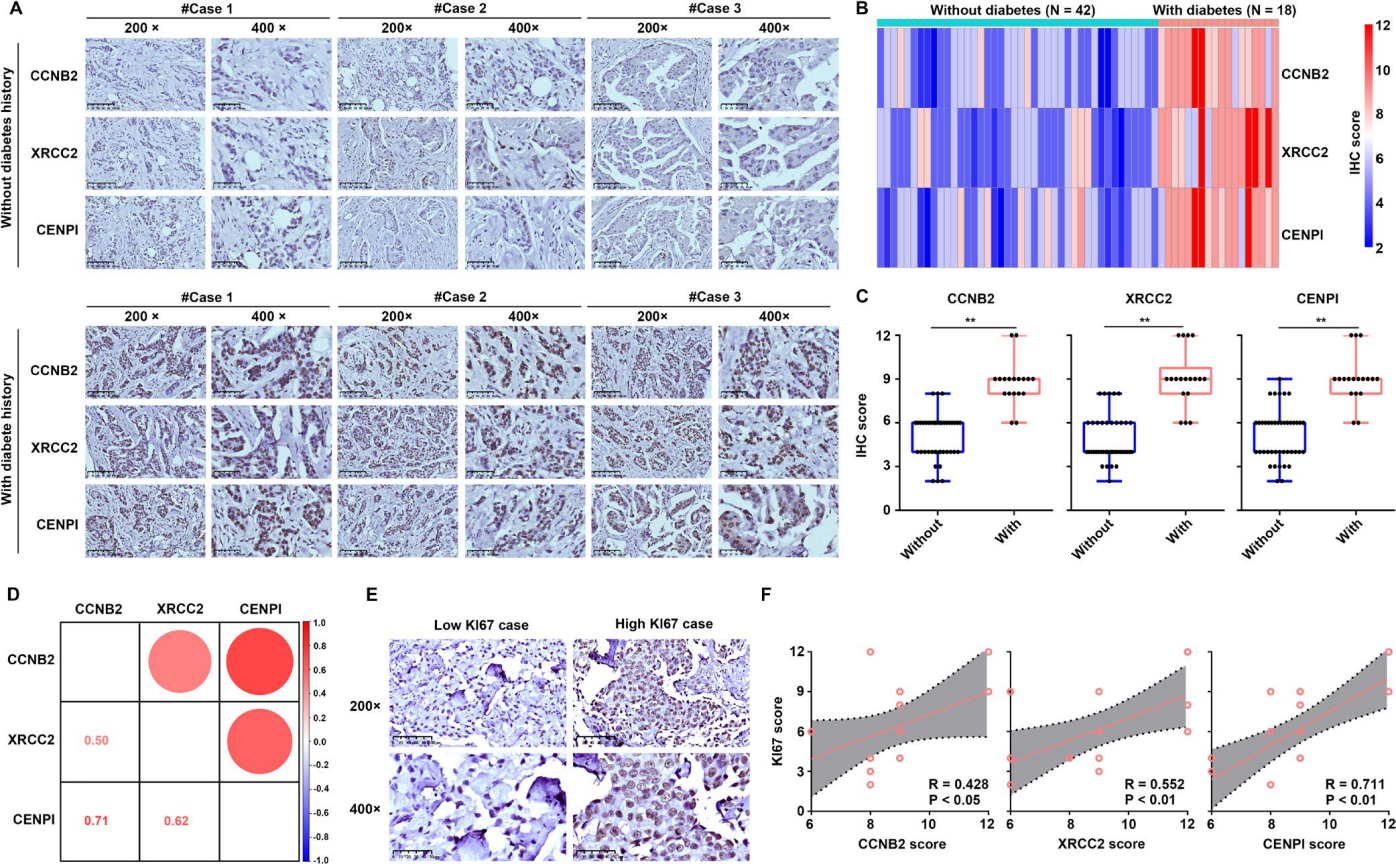
CCNB2, XRCC2, and CENPI were up‐regulated in BC tissues from patients with a diabetes history. (A) Typical immunohistochemical diagram. (B) CCNB2, XRCC2, and CENPI scores for each sample. (C) Expression of CCNB2, XRCC2, and CENPI between BC tissues from patients with and without a diabetes history. (D) Co‐expression relationship between CCNB2, XRCC2, and CENPI in BC tissues. (E) KI67 statin in BC tissues provided by patients with diabetes. (F) Co‐expression relationship between CCNB2, XRCC2, and CENPI for KI67. **p < 0.01.

The expression levels of CCNB2, XRCC2, and CENPI were analyzed in BC tissues obtained from our research cohort. Immunohistochemistry (IHC) analysis was conducted (Figure 7A,B), revealing that CCNB2, XRCC2, and CENPI were significantly upregulated in BC tissues from patients with a history of diabetes compared to those from patients without such a history (Figure 7C). It is worth noting that we found a positive correlation in protein expression between CCNB2, XRCC2, and CENPI in the 60 BC tissues (Figure 7D). Additionally, when examining the expression of KI67 in tissues from BC patients with a history of diabetes (Figure 7E), we observed a positive correlation between the expression of CCNB2, XRCC2, and CENPI and KI67 (Figure 7F).

**FIGURE 8:**
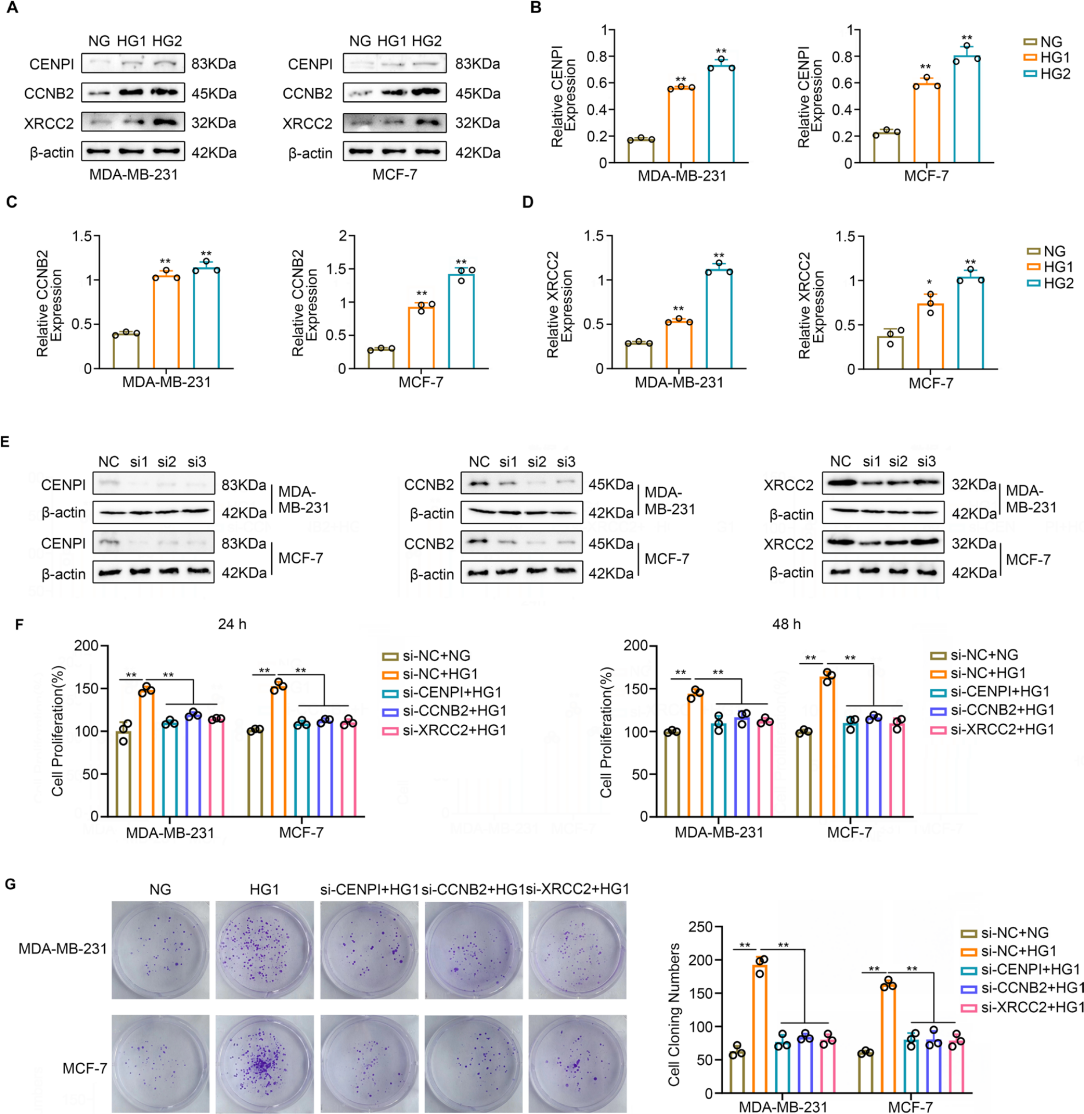
Upregulation in the expression levels of CENPI, CCNB2, and XRCC2 in BC cells cultured under hyperglycemic conditions, while the knockdown of CCNB2, XRCC2, and CENPI potentially attenuates the hyperglycemia effects on BC proliferation. (A) Western blot analysis revealed a significant upregulation in the expression levels of CENPI, CCNB2, and XRCC2 in BC cells cultured under hyperglycemic conditions. (B) CENPI's WB quantitative graph. (C) CCNB2's WB quantitative graph. (D) XRCC2's WB quantitative graph. (E) si1‐CENPI, si2‐CCNB2, and si1‐XRCC2 achieved the highest silencing efficiency. (F) Hyperglycemia enhanced the proliferation of MCF‐7 and MDA‐MB‐231 cells. The knockdown of CENPI, CCNB2, and XRCC2 partially mitigated the proliferative effects induced by hyperglycemia. (G) Hyperglycemia enhanced the colony formation capabilities of MCF‐7 and MDA‐MB‐231 cells. The knockdown of CENPI, CCNB2, and XRCC2 partially mitigated the colony‐forming effects induced by hyperglycemia. *p < 0.05; **p < 0.01.

Hyperglycemia is a critical characteristic of diabetes. Consequently, we treated MCF‐7 and MDA‐MB‐231 cells under both normoglycemic and hyperglycemic conditions. Western blot analysis revealed a significant upregulation in the expression levels of CENPI (Figure 8A,B), CCNB2 (Figure 8A,C), and XRCC2 (Figure 8A,D) in BC cells cultured under hyperglycemic conditions.

Previous studies have suggested that hyperglycemia is a pivotal factor in promoting the progression of BC [29]. Therefore, we investigated whether CENPI, CCNB2, and XRCC2 play a role in the hyperglycemia‐induced progression of BC. siRNAs targeting CENPI, CCNB2, and XRCC2 were employed to generate gene knockdown cell lines. The results demonstrated that si1‐CENPI, si2‐CCNB2, and si1‐XRCC2 achieved the highest silencing efficiency (Figure 8E). Utilizing the CCK‐8 assay (Figure 8F) and colony formation assay (Figure 8G), we observed that hyperglycemia enhanced the proliferation and colony formation capabilities of MCF‐7 and MDA‐MB‐231 cells. However, the knockdown of CENPI, CCNB2, and XRCC2 partially mitigated the proliferative and colony‐forming effects induced by hyperglycemia (Figure 8F,G).

**FIGURE 9:**
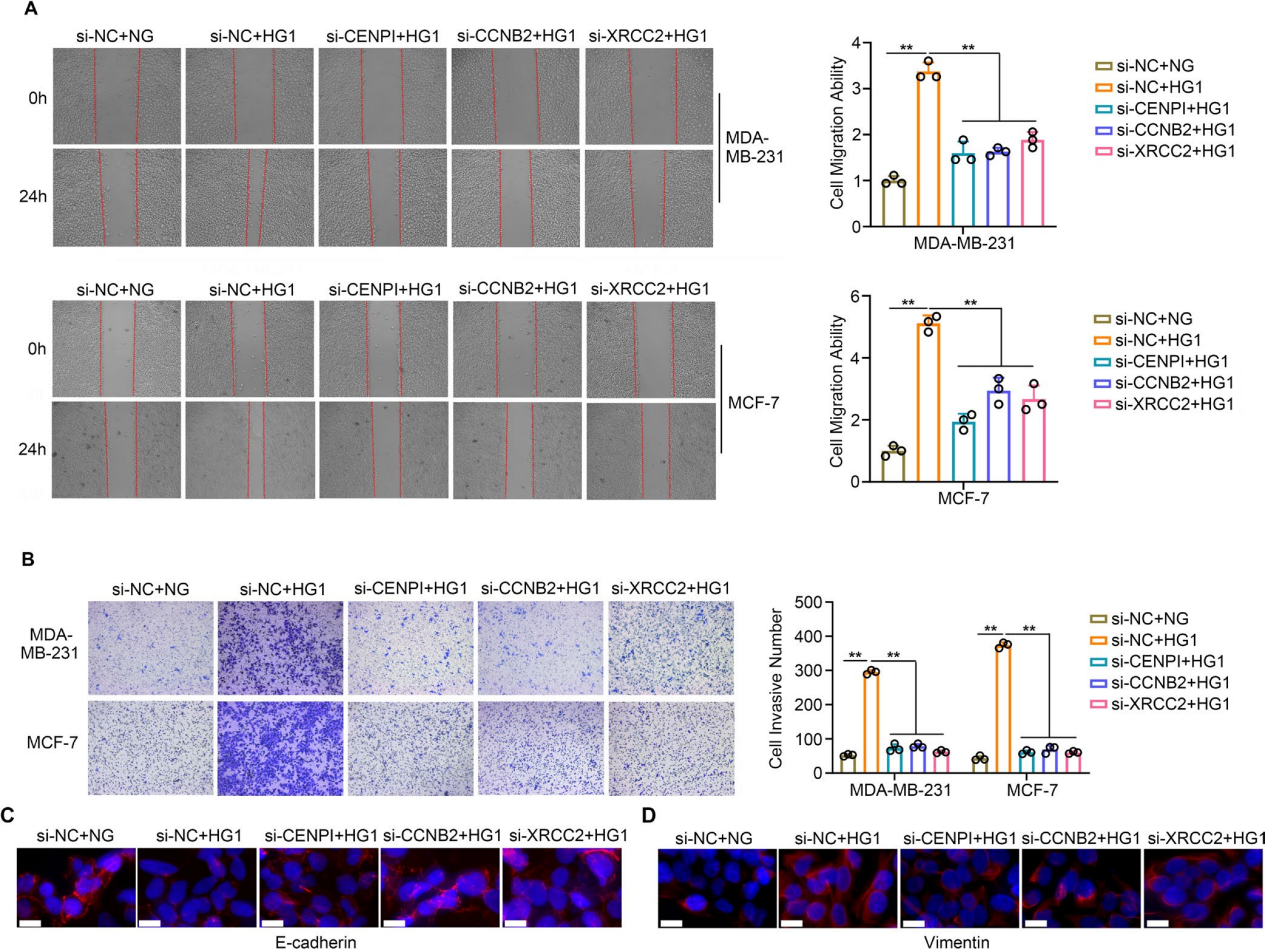
Knockdown of CCNB2, XRCC2, and CENPI potentially attenuate the adverse impacts of T2DM on BC progression. (A) Wound healing assays showed knockdown of CENPI, CCNB2, and XRCC2 partially mitigated the stimulative effects of hyperglycemia on cell migration. (B) Transwell assays showed knockdown of CENPI, CCNB2, and XRCC2 partially mitigated the stimulative effects of hyperglycemia on cell invasiveness. (C) Knockdown of CENPI, CCNB2, and XRCC2 partially increased E‐cadherin in MDA‐MB‐231 cells under hyperglycemia. (D) Knockdown of CENPI, CCNB2, and XRCC2 partially reduced vimentin in MDA‐MB‐231 cells under hyperglycemia. White line means 20 μm. **p < 0.01.

Similarly, through the conduct of wound healing assays (Figure 9A) and transwell assays (Figure 9B), we observed that the knockdown of CENPI, CCNB2, and XRCC2 partially mitigated the stimulative effects of hyperglycemia on cell migration and invasiveness. Furthermore, when we checked the EMT biomarker E‐cadherin (Figure 9C) and vimentin (Figure 9D), we found that reduced E‐cadherin and elevated vimentin were observed in MDA‐MB‐231 cells treated with hyperglycemia, which effects the knockdown of CENPI, CCNB2, and XRCC2 partially mitigated Collectively, these findings suggest that the knockdown of CCNB2, XRCC2, and CENPI may attenuate the deleterious impacts of T2DM on BC progression.

## 4 Discussion

DM and BC are two prevalent chronic diseases that exhibit a significant interrelationship [30]. Epidemiological evidence indicated that diabetes is correlated with an elevated risk of developing BC. Specifically, the detection rate of malignant tumors, including BC, is higher among patients with T2DM [31]. Research has demonstrated that T2DM may elevate the risk of BC in women by approximately two‐ to three‐fold. Furthermore, the prevalence of diabetes among BC patients is on the rise [32]. BC patients exhibited an elevated risk of developing diabetes, with the likelihood being approximately 20% higher 10 years post‐diagnosis compared to age‐matched counterparts [33]. Diabetes not only serves as a risk factor for BC but may also correlate with a poorer prognosis in BC patients. Consequently, overall survival rates may be diminished for BC patients who concurrently have diabetes [34]. These findings indicate a complex interplay between diabetes and BC, underscoring the necessity for further research to elucidate their relationship and to inform both prevention and treatment strategies. This study represents the inaugural investigation into the shared genetic components and common molecular signatures of BC and T2DM utilizing WGCNA and scRNA‐seq analysis, with the aim of enhancing early detection, improving treatment strategies, and facilitating timely prevention.

In the present study, through comprehensive bioinformatics analyses, including WGCNA and DEG analysis, we identified a total of 27 common hub genes shared between T2DM and BC. These genes exhibited significant enrichment in pathways pertinent to cell differentiation, cellular developmental processes, focal adhesion, and the MAPK signaling pathway. Notably, among the 27 genes analyzed, CCNB2, XRCC2, and CENPI were correlated with poor prognosis in BC. Furthermore, single‐cell RNA sequencing analysis demonstrated that CCNB2, XRCC2, and CENPI are predominantly enriched in cancer cells within BC tissues.

Centromere I (CENPI) is a critical protein involved in cellular mitosis, particularly in the precise alignment and segregation of chromosomes. It is a member of the centromerin family and is indispensable for the formation of effective microtubule attachment sites [35]. Notably, CENPI mRNA expression is upregulated in the majority of cancer types and is significantly associated with cancer survival outcomes, indicating its potential as a biomarker for cancer diagnosis and prognosis prediction [36]. Furthermore, the overexpression of CENPI has been shown to enhance chromosomal instability in BC cells and is indicative of poor prognosis in estrogen receptor‐positive BC [37].

Cyclin B2 (CCNB2) serves as a critical regulator of the G2/M transition within the cell cycle [38]. Empirical studies have demonstrated that CCNB2 is overexpressed in various malignancies and is linked to tumor aggressiveness and unfavorable prognosis [39]. Specifically, in BC, elevated CCNB2 expression is correlated with increased lymphovascular invasion [40]. Furthermore, it is associated with tumor enlargement, higher histological grade, hormone receptor negativity, HER2 positivity, and reduced patient survival [41]. Additionally, CCNB2 expression has been positively correlated with the extent of immune cell infiltration in BC [42].

The XRCC2 (X‐ray repair cross‐complementing 2) gene encodes a critical protein involved in the homologous recombination repair of DNA double‐strand breaks. XRCC2 forms the BCDX2 complex with RAD51B, RAD51C, and RAD51D, functioning downstream of BRCA2 and upstream of RAD51. This complex coordinates the assembly of RAD51 on single‐stranded DNA, a process essential for replication fork protection and the repair of double‐strand breaks [43]. Naser et al. proposed that XRCC2 may be classified as an oncogene and that its structural analysis through targeted next‐generation sequencing and expression evaluation could serve as a potential biomarker in BC [44]. Additionally, other studies have demonstrated that mutations in the XRCC2 gene are correlated with an elevated risk of BC [45]. Alongside BRCA1 and BRCA2, XRCC2 is considered to be a significant factor in the genetic susceptibility to BC [46].

However, the mechanisms underlying the up‐regulation of CENPI, CCNB2, and XRCC2 in BC remained inadequately understood. In the present study, we provided novel evidence demonstrating that CENPI, CCNB2, and XRCC2 are associated with the hyperglycemic microenvironment and the biological functions of BC cells. CENPI, CCNB2, and XRCC2 were up‐regulated in BC tissues provided by patients with a diabetes history. Specifically, the expression of CENPI, CCNB2, and XRCC2 was found to be elevated under hyperglycemic conditions. Furthermore, knockdown of CCNB2, XRCC2, and CENPI appeared to mitigate the effects of hyperglycemia on BC cell proliferation and mobility. CCNB2, XRCC2, and CENPI may be pathogenetic link molecules between T2DM and BC, indicating that targeting them may attenuate the deleterious impacts of T2DM on BC progression.

Although our study provides substantial evidence elucidating the molecular mechanisms through which diabetes promotes BC progression, several limitations remain. First, the detailed mechanism by which high glucose upregulates CCNB2, XRCC2, and CENPI is still unclear. Moreover, it remains uncertain whether the in vivo knockdown of CCNB2, XRCC2, and CENPI can reverse the diabetically induced promotion of BC. We plan to employ additional experimental approaches to further investigate these aspects in future research.

## 5 Conclusions

Taken together, through comprehensive bioinformatics analyses, we identified CCNB2, XRCC2, and CENPI as critical pathogenic mediators linking T2DM and BC. Elevated hyperglycemia was observed to upregulate the expression levels of CCNB2, XRCC2, and CENPI in BC cells, correlating with enhanced cell proliferation and mobility. The suppression of these genes partially mitigated the pro‐proliferative and pro‐migratory effects induced by hyperglycemia in BC cells. Consequently, targeting these molecular pathways may offer a therapeutic strategy to attenuate the adverse impacts of T2DM on BC progression.

## Author Contributions

**Xin Bao:** conceptualization (equal), formal analysis (lead), software (equal), validation (lead), visualization (lead), writing – original draft (lead). **Zhirui Zeng:** conceptualization (equal), data curation (lead), methodology (equal), project administration (equal), resources (equal), software (equal), supervision (equal), writing – review and editing (lead). **Wenjing Tang:** formal analysis (equal), investigation (equal), validation (equal). **Dahuan Li:** investigation (equal), validation (equal). **Xianrui Fan:** investigation (equal), validation (equal). **Kang Chen:** investigation (equal), validation (equal). **Yongkang Wang:** investigation (equal), validation (equal). **Weijie Ai:** investigation (equal), validation (equal). **Qian Yang:** investigation (equal), validation (equal). **Shu Liu:** project administration (equal), supervision (equal). **Tengxiang Chen:** funding acquisition (equal), project administration (equal), resources (equal), supervision (equal).

## Ethics Statement

A total of 60 BC tissues were collected from the Affiliated Hospital of Guizhou Medical University, following approval by the Human Ethics Committee of the same institution (Approval number: 2024‐187). Patients were consented by an informed consent process that was reviewed by the Human Ethics Committee of the Affiliated Hospital of Guizhou Medical University, and the ethics committeecertified that the study was performed in accordance with the ethical standards as laid down in the Declaration of Helsinki. All of the public databases in the current study were openly available, and the Human Ethics Committee of the Affiliated Hospital of Guizhou Medical University waived the need for ethics approval and the need to obtain consent.

## Consent

The authors have nothing to report.

## Conflicts of Interest

The authors declare no conflicts of interest.

## Supporting information


**Figure S1.** Modules whose GS values positively correlated with their respective MM values except blue module in T2DM. (A) Turquoise module. (B) Red module. (C) Green module. (D) Brown module.
**Figure S2.** Modules whose GS values positively correlated with their respective MM values except turquoise module in BC. (A) Skyblue module. (B) Lightyellow module. (C) Darkred module. (D) Cyan module.
**Table S1.** Genes within the five significative modules exhibiting |MM| ≥ 0.5 and |GS| ≥ 0.5 were designated as core genes of T2DM and subjected to further analysis.
**Table S2.** Genes within the significative modules exhibiting |MM| ≥ 0.5 and |GS| ≥ 0.5 were designated as core module genes of BC and were selected for further analysis.
**Table S3.** The expression of CCNB2, XRCC2, and CENPI was elevated in both T2DM and BRCA samples.

## Data Availability

The corresponding author can be contacted for raw data if necessary.
