# Supplementary material for: Bioinformatics Combined With Biological Experiments to Identify the Pathogenetic Link of Type 2 Diabetes for Breast Cancer
Source: Cancer Med. 2025 Apr 9;14(7):e70759. doi: 10.1002/cam4.70759 (PMC11979791; doi:10.1002/cam4.70759)
Supplement: Supplementary file 1 — Figure S1. Modules whose GS values positively correlated with their respective MM values except blue module in T2DM. (A) Turquoise module. (B) Red module. (C) Green module. (D) Brown module. Figure S2. Modules whose GS values positively correlated with their respective MM values except turquoise module in BC. (A) Skyblue module. (B) Lightyellow module. (C) Darkred module. (D) Cyan module. Table S1. Genes within the five significative modules exhibiting |MM| ≥ 0.5 and |GS| ≥ 0.5 were designated as core genes of T2DM and subjected to further analysis. Table S2. Genes within the significative modules exhibiting |MM| ≥ 0.5 and |GS| ≥ 0.5 were designated as core module genes of BC and were selected for further analysis. Table S3. The expression of CCNB2, XRCC2, and CENPI was elevated in both T2DM and BRCA samples. [file CAM4-14-e70759-s001.zip › cam470759-sup-0006-Supplementary Table 3.docx]

| ID | BRCA(logFC) | T2DM(logFC) |
| --- | --- | --- |
| CCNB2 | 3.301110588 | 0.375652796 |
| XRCC2 | 1.53720902 | 0.44962533 |
| CENPI | 2.206617244 | 0.790559307 |
